# Air Temperature and Humidity at the Bottom of Desert Wolf Spider Burrows Are Not Affected by Surface Conditions

**DOI:** 10.3390/insects12100943

**Published:** 2021-10-18

**Authors:** Irene Steves, Pedro Berliner, Berry Pinshow

**Affiliations:** 1 Mitrani Department of Desert Ecology, Jacob Blaustein Institutes for Desert Research, Ben-Gurion University of the Negev, Midreshet Ben-Gurion 8499000, Israel; irene.steves@gmail.com; 2 French Associates Institute for Agriculture and Biotechnology, Jacob Blaustein Institutes for Desert Research, Ben-Gurion University of the Negev, Midreshet Ben-Gurion 8499000, Israel; berliner@bgu.ac.il

**Keywords:** Lycosidae, spider burrows, activity patterns, responses to disturbance, microenvironment

## Abstract

**Simple Summary:**

We investigated whether trapdoors, which small Negev Desert wolf spiders (*Lycosa* sp.) use to close their burrows, serve to maintain favorable conditions of temperature and humidity within them. We removed trapdoors from burrow entrances and monitored changes in temperature and relative humidity in their bottoms, where the spiders reside during the day. We also followed the behavioral responses of these spiders to trapdoor removal at different times of the day and in different seasons and monitored temperature and relative humidity in artificial burrows during summer mornings and at midday. At noon, air temperature at the bottom of open artificial burrows increased by less than 1 °C more than in covered ones, and total humidity remained constant, even though air temperature at the soil surface reached 55 °C when the burrow temperature was 35 °C. The relatively small increase in air temperature in uncovered burrows at midday was probably due to the penetration of direct solar radiation. Thus, it is evident that the presence of a trapdoor has a negligible effect on the microclimate at the bottom of these spiders’ burrows and its roles are more likely predator avoidance and prevention of flooding and stones and sand from falling in.

**Abstract:**

Burrows are animal-built structures that can buffer their occupants against the vagaries of the weather and provide protection from predators. We investigated whether the trapdoors of wolf spider (Lycosa sp.; temporary working name *"L. hyraculus"*) burrows in the Negev Desert serve to maintain favorable environmental conditions within the burrow by removing trapdoors and monitoring the ensuing temperature and relative humidity regime within them. We also monitored the behavioral responses of “*L. hyraculus*” to trapdoor removal at different times of the day and in different seasons. “*L. hyraculus*” often spun silk mesh in their burrow entrances in response to trapdoor removal during the day, possibly to deter diurnal predators. The frequency of web-spinning peaked on summer mornings, but spiders began spinning webs sooner after trapdoor removal later in the day. In addition, we monitored temperature and relative humidity in artificial burrows in the summer during the morning and at midday. At noon, air temperature (*T*_a_) at the bottom of open burrows increased by <1 °C more than in covered burrows, but water vapor pressure in burrows did not change. The relatively small increase in *T*_a_ in uncovered burrows at midday can probably be ascribed to the penetration of direct solar radiation. Thus, air temperature and humidity at the bottom of the burrow are apparently decoupled from airflow at the surface.

## 1. Introduction

Animal-built structures vary widely in shape and size and have evolved through natural selection on their occupants [1]. Burrows protect their occupants from predators and parasites [2], provide shelter during periods of vulnerability, such as during molting or when rearing young [3], and buffer their occupants from the vagaries of the environment, including from events like fire, flooding, and rain [4,5]. Also, these structures can serve as adaptive interfaces for managing fluxes of gases and energy between the inhabitant(s) and their physical environment [1,6]. Burrows are ubiquitous structures, and, on land, are dug by animals whose size ranges from small arthropods (e.g., ants and spiders) to large mammals (e.g., aardvarks and warthogs) [7]. In arid environments such as deserts, they may protect their inhabitants from desiccation and thermal stress [8].

The physical isolation of an animal in its burrow could potentially result in the depletion or accumulation of respiratory or other gases (O_2_, CO_2_, NH_3_, etc.) due to limited ventilation and thus affect its normal physiological functions. This is, however, not necessarily the case, and several ventilation mechanisms have been reported in the literature for relatively large bi-entrance [6,9] and single entrance burrows [10,11,12].

It has been proposed that forced ventilation of the lower parts of their burrows may be detrimental to ectothermic animals, as implied from the fact that some scorpions build burrows in a manner that reduces gas exchange [12,13], and many spider species directly occlude ventilation by capping the entrances of their burrows. An example of the latter is species of burrowing spiders that cover the entrances with trapdoors. The role of trapdoors in maintaining favorable thermal and hydric conditions within the burrow is indirectly supported by studies of spider behavior in arid environments. For example, *Nemesia caementaria* (Latreille, 1799) seal their burrows during estivation [14], presumably to maintain both cool and humid conditions. Also, *Aliatypus* (Smith, 1908) trapdoor spiders that inhabit arid areas in western North America were found to seal their burrows more often than their conspecifics that live in more humid conditions [15,16,17].

None of the above-mentioned studies, however, are supported by direct measurements of the relevant microclimatic variables in the field. Our aim was therefore to quantitatively assess the effects of the presence or absence of a trapdoor on the environmental conditions at the bottom of burrows of a common, but undescribed, lycosid spider species. To this end, we made detailed measurements in a set of artificial burrows, built in the field to resemble natural ones as closely as possible; we also made observations of spider behavior in their natural burrows after selectively removing trapdoors at different times of the day.

Our working hypotheses were that the presence of trapdoors would have measurable effects on air temperature (*T*_a_) and relative humidity (RH) in the burrows and that their removal would affect environmental conditions at the bottom of the burrow and therefore induce spider responses depending on the prevailing environmental conditions at the surface. Specifically, we predicted that warmer and drier surface conditions would elicit faster responses to trapdoor removal.

## 2. Materials and Methods

Field site—Our research site was a 3-hectare plot on the Sede Zin plateau in the Negev Desert highlands, less than 50 m from the north-east corner of the Sede Boqer Campus of Ben-Gurion University of the Negev at Midreshet Ben-Gurion, Israel (30°51′38″ N, 34°46′40″ E), where the Jacob Blaustein Institutes for Desert Research (BIDR) meteorology station is located. The station provides hourly-averaged values of standard environmental variables. The loess soil in the study area is sparsely populated by shrubs, dominated by *Hammada scoparia* (Pomel) Iljin.

Study species—The many groups of spiders that build burrows include wolf spiders (reviewed in [18]). We studied a trapdoor-building wolf spider (Lycosidae), that, although is locally abundant, is yet undescribed in the scientific literature. However, in March 2017, it was given the temporary working name, “*Lycosa hyraculus”* (Igor Armiach Steinpress and colleagues, National Arachnid Collection, The Hebrew University of Jerusalem, personal communication) that we have used below. All the spiders studied were adults and we did not differentiate among sexes.

During the day, “*L. hyraculus”* generally remain in their burrows with the trapdoor closed. At night, they actively hunt on the soil surface within the vicinity of their burrows, typically less than 1 m away, and the trapdoors are left open (personal observations). Like other lycosids, “*L. hyraculus”* is a generalist predator and we observed them capturing or holding moths, beetles, termites, scorpions, smaller spiders, and other arthropods.

**Figure 1:**
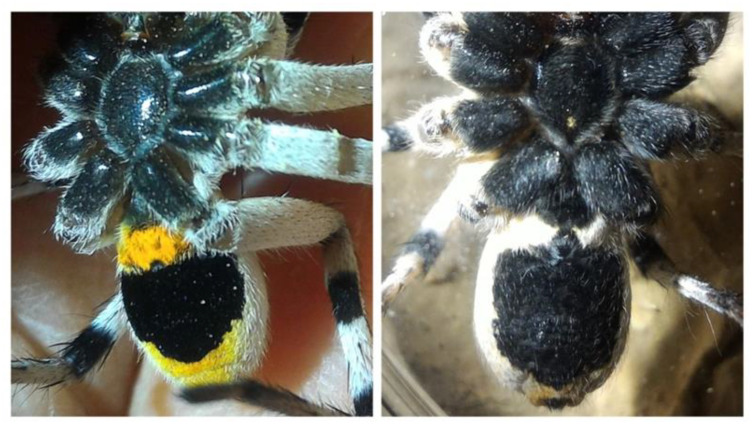
Morphological differences between the spiders, Lycosa olivieri and “*L. hyraculus"*. The two spiders can be distinguished by the coloration on the abdomen of subadults and adults, as well as by their burrow structure. *L. olivieri* (**left**) often builds a grass turret around its burrow entrance and has extensive orange coloration on its abdomen, whereas the trapdoor-building “*L. hyraculus"* (**right**) is only orange around its spinnerets.

“*Lycosa hyraculus*” co-occurs with *Lycosa olivieri* Simon, 1876, also a widely distributed species, but the two differ in their coloration and the construction of their burrow entrances. *Lycosa hyraculus* covers and camouflages its burrow entrance, usually with a trapdoor made from a piece of soil crust. *L. olivieri*, in contrast, builds a grass or soil turret around its burrow entrance. The presence or absence of a turret was found to be a reliable species-specific indicator in other burrowing wolf spiders [19]. Individuals of the two species can also be distinguished by the extent of the orange coloration on their abdomens, which is found only faintly around the spinnerets in “*L. hyraculus*” but extends towards the cephalothorax in *L. olivieri* (Figure 1).

Natural burrow structure: We found that “*L. hyraculus"* builds simple, vertical burrows that are between 49 and 145 mm deep (mean = 101.5 mm, SD = 20.0 mm, *n* = 196) and between 7 and 21 mm in diameter (mean = 11.89 mm, SD = 2.56 mm, *n* = 196). Ninety-eight percent of the burrow entrance diameters were less than 17 mm. Diameters and depths were linearly correlated (diameter (mm) = 4.51 + 0.073 × depth (mm); r^2^ = 0.3228; F = 92.47; *p* = 0.0001). Burrows are lined with a layer of silk, which may stabilize the soil [16] and facilitate the movement of the spider inside [20]. Like other trapdoor-building spiders, “*L. hyraculus"* constructs the trapdoor from materials available in its environment, most often pieces of soil crust, but also small pebbles and snail shells, and attaches it to the burrow entrance with silk strands [15,21]. The undersides of “*L. hyraculus"* trapdoors are usually lined with silk.

Natural burrow survey: At night, we found the spiders visually from reflections of their *tapeta lucida* in our headlamp beams. We located over 200 “*L. hyraculus*” and their burrows between May 2016 and July 2017, recorded the GPS location (MAP-330, Magellan, San Dimas, California) of each and marked it with a flag and identification number. We measured burrow entrance diameters and spider body lengths (from the anterior end of the cephalothorax to the posterior end of the abdomen) using digital calipers (Absolute Digimatic, Mituyo, Kawasaki, Japan). The spiders remained immobile on the soil surface near their burrow entrances in our headlamp beams and we could lay the caliper next to them to measure body length. Depth was manually measured with a thin rod against a steel ruler.

Artificial burrows: We made artificial burrows of two diameters that we chose to represent the range of natural burrow dimensions (cylinders with a diameter of 11 or 17 mm, which bracket the range of natural burrow diameters and 100 mm deep, the mean depth of natural burrows) to standardize burrow shape and size. To make a burrow, we first slightly wetted the soil surface to stabilize the soil crust. We then drove a hollow pipe (1 mm thick, 10 mm, or 16 mm external diameter) 100 mm into the ground, and slowly twisted it up to extract soil. We fitted the burrows with sensors and immediately covered them with opaque, soil-colored plaster discs (Glastone Dental Stone, DENTSPLY International, York, PA, USA), 10–20 mm in diameter and 2–3 mm thick, to mimic natural cover and to prevent the soil from drying out at the bottom of the burrow. The covered burrows were allowed to equilibrate with the surrounding soil at least 24 h before measurements were made.

Measurements: (1) Temperature: We measured *T*_a_ in ten artificial burrows of each size (11 mm or 17 mm diameter,) twice a day (early morning and midday) on six days in June 2017. Each burrow was fitted with a type-T thermocouple (24 SWG, 0.511 mm diameter) at the bottom of the burrow with the junctions bent upwards to ensure that they did not touch the bottom or sides. The thus measured air temperatures are indicative of the environment in which spiders likely reside on hot days [22]. We concurrently measured *T*_a_ 10 mm above the soil surface with a thermocouple shaded by a white polyurethane board. All thermocouples were connected to dataloggers (CR23X Micrologger, Campbell Scientific, Inc., Logan, UT, USA), which recorded *T*_a_ at 10 s intervals and averaged once per minute.

(2) Relative humidity: We measured relative humidity (RH) and temperature simultaneously using compact probes (5.6 mm diameter, USB-TRH300, Dracal Technologies, Brossard, Canada), whose overall accuracy was at 25 °C is ±2% RH. We calibrated the probes against a humidity sensor conforming to the Israel Meteorological Service standard (HC2-S3 humidity sensor, Rotronic AG, Bassersdorf, Switzerland) at the BIDR meteorology station for 24 h on 30 May and 8 August 2017.

Since RH depends on temperature, we examined changes in the water content of the air at the bottom of the burrow by calculating the water vapor pressure (*e*). We first solved for the saturation water vapor pressure (*e*_s_, hPa) at the given temperature (using Equations (2.5) and (2.6) of [23] and calculated actual vapor pressure *e*, as *e = RH* × *e*_s_.

**Figure 2:**
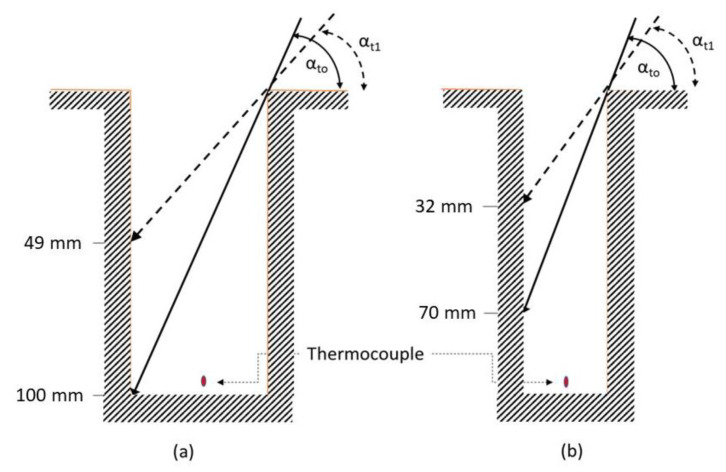
Schematic diagram depicting maximum direct radiation penetration into vertical, cylindrical burrows with diameters of 17 mm (**a**) and 11 mm (**b**). During the three measurement days (16, 17, and 18 June) described in the text. Computed sun elevation angles immediately after removal of trapdoor (α_to_) and before replacement (α_t1_).

(3) Estimation of solar radiation penetration: The thermal and hydric conditions in the burrow may be affected by diffuse and direct radiation entering open burrows. Diffuse radiation from the celestial hemisphere that enters the burrow can be neglected because the solid angle subtended by the burrow’s entrance is extremely small. Direct radiation penetration into the burrow depends, however, on the elevation of the sun and the size of the burrow opening. Assuming a straight, vertical burrow, the maximum depth that direct radiation could penetrate the burrow (*d*_rad_) is:

$$d_{\mathrm{rad}}=D\;\times tan\left(\alpha \right)$$

where *D* is the burrow diameter and *α* is the solar elevation angle. We calculated the elevation angles for the time of opening and closing of the burrows at midday using the NOAA Solar Position Calculator (https://www.esrl.noaa.gov/gmd/grad/solcalc/) (accessed on 1 September 2021). Typical values were 81° and 71° at the beginning and end of the measurement trial (13:00–14:00) during the period of 14–18 June 2017 (Figure 2). We also calculated *d*_rad_ for the diameters of the natural burrow and compared them to their corresponding depths (*n* = 181). Thus, we could determine if and for how long direct radiation reached the bottom of the natural “*L. hyraculus"* burrows.

Field trials in artificial burrows: Trial (1) On each trial day, we randomly chose three burrows and opened them for one hour at 07:00, and then three different burrows were opened for one hour at 13:00, leaving four burrows with trapdoors untouched as controls. Opened burrows were closed after each trial. Trials were repeated on three different days for both large (17 mm diameter) and medium-sized (11 mm) burrows. Trial (2) We monitored RH on the 30 July 2017, only at the bottom of the large (17 mm diameter) burrows due to the size of the sensors. We measured RH and temperature simultaneously in six burrows (three of them randomly uncovered) at 1-minute intervals, for 1 h (13:00–14:00), for at least 15 min before and after uncovering. Each set of trials (early morning or midday, mid-sized or large burrows) was analyzed separately. Opened burrows were closed after each trial.

Spider responses to trapdoor removal: We examined changes in “*L. hyraculus"* responses to trapdoor removal at different times of the day and in different seasons. For each trial, we removed the trapdoor and monitored the response of the spider for up to 24 h.

Seasonal patterns: We compared spider responses to trapdoor removal during the summer (June 2016, *n* = 16; July 2017, *n* = 33) and the winter (January 2017, *n* = 18, 30, or 34, depending on the day). For each trial day, we removed half of the trapdoors in the early morning (06:00) and half at midday (12:00). We monitored burrows at 1-hour intervals for 3 h following trapdoor removal and at 3-hour intervals thereafter until sundown. We defined *t*_web,h_ as the time in hours from trapdoor removal until when we first saw a complete mesh in the burrow entrance. If a spider partially covered its burrow entrance but never completed it, we assumed that we had disturbed the process during our examination and noted the time of completion rounded up to the next full hour. We also used data collected at the BIDR meteorology station to relate changes in spider responses to soil and air temperatures.

Daily patterns: We examined changes in response time by taking series of time-lapse photographs of spiders at their burrow entrances with a camera (SP-570UZ, Olympus, Tokyo) positioned on a short tripod above and to the north of a burrow entrance to avoid shadows and set the camera to take 100 photographs at 1-minute intervals. The first picture was taken with the burrow covered and a ruler next to the trapdoor. Thereafter, we carefully removed the burrow trapdoor and left the area. We moved the camera to a different burrow every 2 h, starting at 07:00 and ending at 19:00 over 15 days between 6 April and 5 May 2017. Since creating the mesh often involved several bouts of activity, it was difficult to define exactly when the repair behavior ended. Thus, we defined *t*_web,m_ as the time in minutes from trapdoor removal to the beginning of mesh-building, when either silk strands or a spider’s spinnerets first became visible at the burrow entrance.

Data analysis of daily spider activity patterns: For each experiment, we compared mean *t*_web_ among groups using one-way ANOVA [24]. We tested the assumptions of normality of distribution and homoscedasticity using the Shapiro–Wilk test and F test, respectively. For group means that were significantly different, we followed the ANOVA with a Tukey post-hoc test.

We examined differences in the fraction of spiders that spun meshes among groups using chi-square analyses. To determine whether and how this fraction changes over the course of a day, we ran a test of equal proportions followed by a chi-squared test for trends in proportions [24]. We also used the Welch–Satterthwaite *t*-test for unequal variances, where necessary. In our comparison between seasons, we ran a post-hoc test with a Bonferroni correction for multiple comparisons after chi-squared analysis [25].

## 3. Results and Discussion

### 3.1. Simulated Burrow Thermal and Hydric Environment

No differences between the temperature measured at the bottom of open and closed burrows, narrow or wide, were observed for the morning measurements (data not presented). The noon burrow-bottom temperature measurement of the wider burrows, however, showed a clear pattern.

We defined Δ*T*_t_ as:

$$\Delta Tt_{\;}=\bar{\left(T_{bi}^{t}-T_{bi}^{0}\right)}-\bar{\left(T_{bck}^{t}-T_{bck}^{0}\right)}$$

where

*T* = temperature (°C)

Superscripted “*t*” denotes = measurement time

Superscripted “0” denotes reference time (five minutes before uncovering)

Subscripted “*bi*” = uncovered burrow i (i = 1 to 3)

Subscripted “*bck*” = covered burrow i (k = 1 to 4)

**Figure 3:**
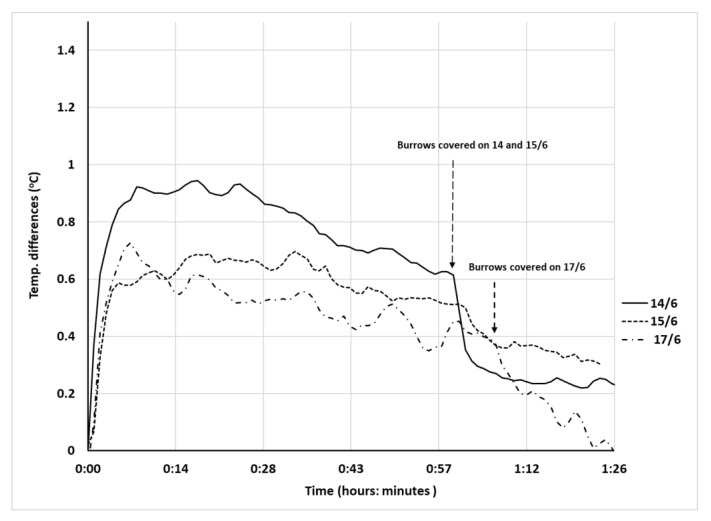
Differences between averaged temperature differences (Δ*T*_t_, see text for definition) in °C of open and closed *Lycosa hyraculus* burrows over three dates (14/6, 15/6, and 17/6) as a function of time elapsed after door removal. Error bars are omitted for clarity.

Δ*T*_t_ data are presented in Figure 3 where a steep increase in Δ*T*_diff_ during the first seven minutes after uncovering can be observed. This rise is followed by a moderate decrease in Δ*T*_t_ and a sharper drop upon re-covering the burrows 60 min after uncovering. To assess the significance of these observations, we analyzed the data for two critical times: 10 min and 60 min after uncovering. The temperature at the bottom of each burrow was measured five minutes before uncovering; this measurement was used as a reference temperature to reduce the effect of the between-burrow temperature variability in our calculations. We compared the changes in the burrow-bottom air temperature of each burrow to its corresponding reference temperature and analyzed the treatment effect 10 and 60 min after the removal of the doors.

Ten minutes after uncovering, the air temperatures at the bottom of the open burrows were higher than those of closed ones for the 14, 15, and 17 of June, respectively (two-tailed *t*-tests for 14/6: mean difference = 0.9042 ± 0.2172 SD, t = 5.45, df = 5, *p* < 0.001; for 15/6: mean difference = 0.5058 ± 0.1031 SD, t = 6.42, df = 5, *p* < 0.001; and for 17/6, Welch–Satterthwaite t-test: mean difference = 0.5558 ± 0.1023 SD, t = 5.99, df = 2.1, *p* < 0.013). Sixty minutes after uncovering and immediately prior to covering the burrows, the mean differences were smaller (two-tailed *t*-tests for 14/6: mean difference = 0.3075, SD = ± 0.1577, *p* < 0.03; for 15/6: mean difference = 0.4475, SD = ± 0.1427, *p* < 0.005; for 17/6, Welch–Satterwhite *t*-test: mean difference = 0.3650, SD = 0.1852, *p* < 0.02). Upon trapdoor removal, direct radiation penetrated into the burrow and illuminated the northern portion of the wall, from the surface down to the bottom, but never illuminating the bottom itself (Trial 1, Figure 2). Therefore, the thermocouple did not intercept direct solar radiation, but intercepted reflected solar radiation from the northern wall and the long wave radiation emitted by the surrounding walls; the illuminated part of the latter emitted a higher flux than the non-illuminated part.

Average burrow-bottom temperatures immediately before uncovering ranged from 33 to 35 °C and, although the temperature differences between uncovered and covered artificial burrows were statistically significant, they were small in magnitude (<1 °C), particularly when compared to soil surface temperatures, which reached 55 °C and 39 °C 100 mm above the soil surface.

Although RH decreased and *T*_a_ increased as expected after uncovering burrows, water vapor pressure differences between covered and uncovered burrows were not significant (data not presented). The fact that both water vapor pressure and temperature appear to be decoupled from the above-ground air flow indicates that resonant mixing or eddy penetration [6,10] are not relevant mechanisms in these burrows. Furthermore, the changes of *T*_a_ in the artificial burrows probably represent the maximum effect of the trapdoor in 10-millimeter deep burrows because natural burrows typically have narrower entrances and because there was no forced ventilation in the bottom of the burrow. Thus, the fact that both water vapor pressure and temperature were not affected by above-ground atmospheric conditions strengthens our conclusion that changes in air temperature at the bottom of the burrow when uncovered were negligible, i.e., the microclimatic conditions at the bottom of the burrows is effectively decoupled from the above-ground airflow.

### 3.2. Expected Solar Radiation Penetration in Natural Burrows

**Figure 4:**
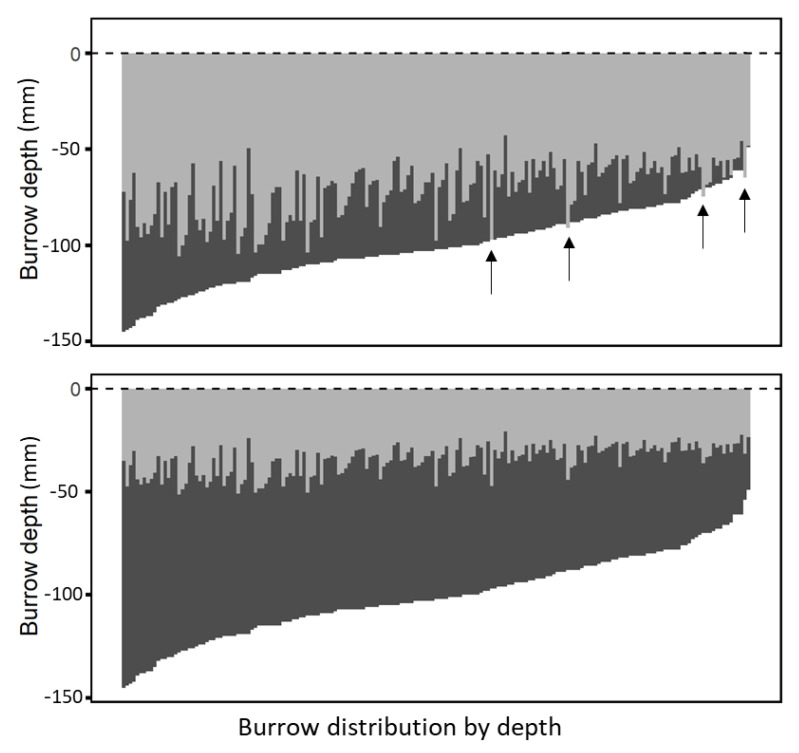
Theoretical maximum depth of direct solar radiation that would penetrate natural *Lycosa hyraculus* burrows at midday on a typical summer day in the Negev Desert Highlands.

Direct radiation calculated from the dimensions of 181 natural burrows reached a maximum of 42% of total burrow depth at 13:00 on a typical summer day (Figure 4). In only four cases, direct radiation would have reached the bottom of the burrow if it was a vertical and straight burrow. One hour later, at 14:00, direct radiation penetrated to 20–58% of the total burrow depth. Therefore, only the upper reaches of the burrow received direct solar radiation during the one-hour exposure. We made no direct measurements, but the temperature of the exposed wall certainly increased and could result in an additional downward flux of sensible heat by conduction in the soil, eventually reaching the lower end of the burrow. We estimate however that this flux is small when compared to the downward flux from the natural soil surface that is continually exposed to solar radiation and does therefore probably not affect the surface temperature of the bottom of the burrow.

Burrows are ordered by depth. Penetration depth (light gray) was calculated using measured spider burrows diameters (*n* = 181) and corresponding depths for solar zenith angles at 13:00 (9.3°, top) and at 14:00 (18.6°, bottom) on 24 June 2017. The length of each light grey line represents the depth of solar radiation penetration, and the lower boundary of the dark grey line corresponds to burrow depth. Burrows marked with arrows would receive direct radiation at the bottom of the burrow at 13:00 local time.

### 3.3. Spider Responses to Trapdoor Removal

**Figure 5:**
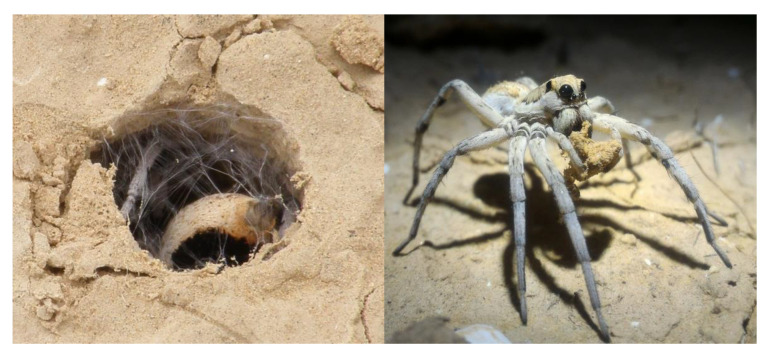
Typical responses of *Lycosa hyraculus* to trapdoor removal. A spider spins a mesh in its burrow entrance (**left**); a spider carries a piece of soil in its pedipalps and chelicerae, possibly to replace a trapdoor (**right**). Spiders may also carry soil during burrow maintenance when they remove accumulated soil by binding it into balls with silk.

Spiders had three main responses to trapdoor removal: (1) no visible change or activity; (2) spinning a mesh in the burrow entrance during the day (Figure 5); or (3) sometimes replacing the trapdoor at night. In fact, when we removed trapdoors from the burrows of “*L. hyraculus,"* the spiders usually emerged at night and replaced their trapdoors with pieces of soil crust. In this way, burrow entrances were usually covered again within 24 h of trapdoor removal. Other trapdoor-building spiders have been observed replacing trapdoors within 2–12 h, but the time of day was not noted [16,26].

The spiders appeared to manipulate the soil crust with their chelicera and by adding silk to fit the burrow entrance, rather than build a new door from soil and silk, as do other spiders [17]. However, we observed that occasionally floppy trapdoors appeared to be held together primarily by silk.

**Figure 6:**
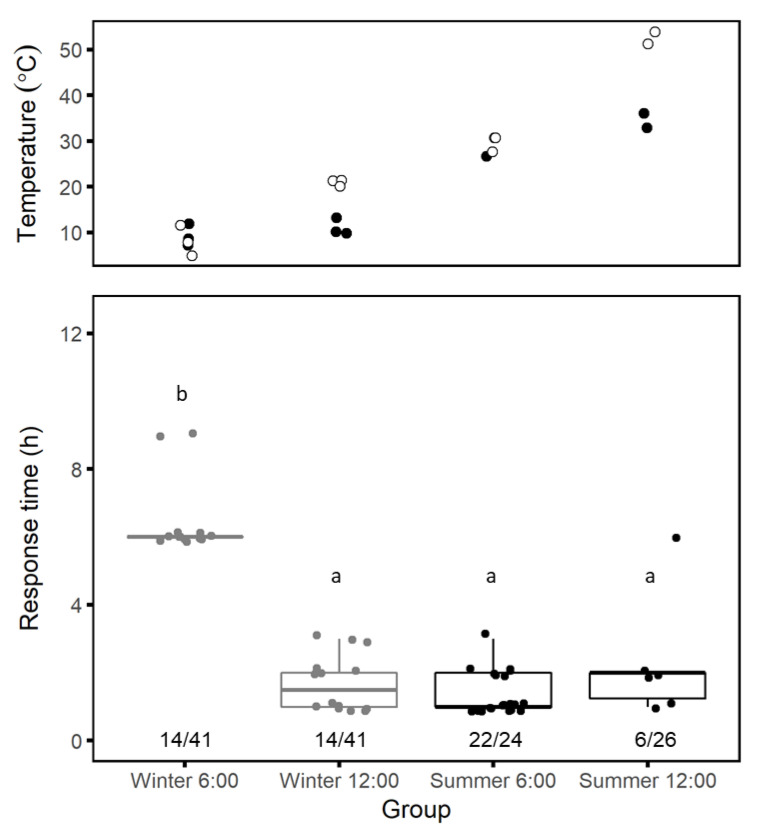
Soil temperatures (**top**) and *Lycosa hyraculus* response time to trapdoor removal (**bottom**) at different times of day and in different seasons (winter in gray, summer in black). Soil surface temperatures (white) and temperatures at 100 mm depth (black) at the beginning of each trial were measured at the Jacob Blaustein Institutes for Desert Research meteorology station. We defined response time, t_web,h_, as the time in hours from when we removed a trapdoor to when we first observed a mesh in the burrow entrance. The fraction of spiders that spun silk in their burrow entrances out of all spiders whose trapdoors were removed is indicated below the boxplots. Individual data points are overlaid on the boxplot to better visualize variance. The thick horizontal line is the median, and the lower and upper hinges correspond to the 1st and 3rd quartiles (overlaid by the median in some cases here). The whiskers extend to the farthest value within 1.5 times the inter-quartile range from the median. Groups with different letters are significantly different (Tukey’s post-hoc test, *p* < 0.05).

Seasonal patterns: Spiders responded to trapdoor removal slowly on winter mornings, with six hours passing, on average, before beginning to spin a mesh in the burrow entrance (one-way ANOVA *F*_3_ = 85.5, *p* < 0.001; Tukey post-hoc *p* < 0.001 for all comparisons against winter morning group; Figure 6). In contrast, in other seasons, most spiders started to spin meshes within 1–2 h of trapdoor removal. Since spiders are ectothermic, the delay in response on winter mornings may be due to cold soil surface temperatures, which in winter are lowest in the early morning (~07:00, ~4–12 °C, BIDR meteorology station).

More *"L. hyraculus"* spun meshes in response to trapdoor removal on summer mornings than at other times ($\chi_{3}^{2}$ = 31.4, *p* < 0.001; post-hoc *p* < 0.001 for all comparisons against the summer morning group, Figure 6). The greater number of individuals responding on summer mornings may be due to their increased activity at higher soil temperatures. However, the response rate also dropped at midday in summer, when soil surface temperatures reached between 33–54 °C, compared to 25–31 °C on summer mornings.

**Figure 7:**
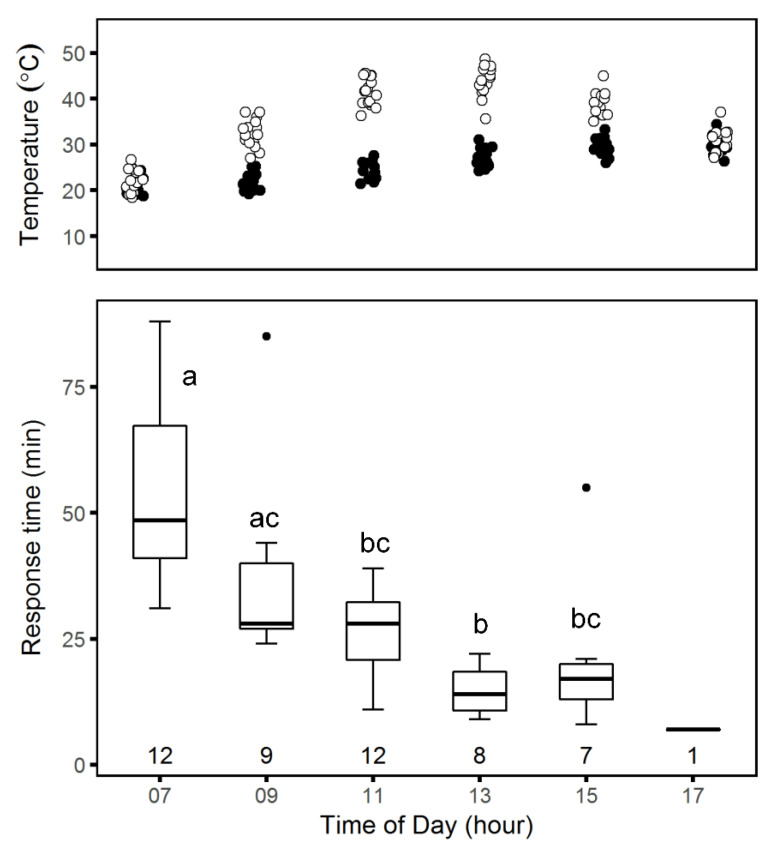
Soil temperatures (**top**) and boxplot of *Lycosa hyraculus* response times to trapdoor removal during the day (**bottom**). Soil surface temperatures (white) and temperatures at 100-millimeter depth (black) at the beginning of each trial were measured at the Jacob Blaustein Institutes for Desert Research meteorology station. Response time (t_web,m_) was defined as the time in minutes from when we removed the trapdoor until the spider began spinning a mesh. Under each boxplot is the number of spiders, of the 15 observed, that spun meshes in that period. The thick horizontal line is the median, and the lower and upper hinges correspond to the 1st and 3rd quartiles. The whiskers extend to the farthest value within 1.5 times the inter-quartile range from the median. Any points beyond the whiskers are plotted individually as outliers. Bars with the same letters are not significantly different (Tukey’s post-hoc test, *p* < 0.05); t_web,m_ is longest in the morning, but fewer spiders spin meshes in the late afternoon.

*Daily patterns:* We tested spider responses to trapdoor removal at different times of the day in the summer, when environmental conditions differed considerably, and found that mean response times were dependent on the time of day (one-way ANOVA, *F*_5_ = 10.1, *p* < 0.001). Response times were significantly longer in the morning (Tukey post-hoc test, *p* < 0.05), with a decreasing trend over the course of the day. As with the seasonal responses, decreases in response time were associated with increases in temperatures in the upper layer of the soil burrow (~100-milimeter deep). The fraction of spiders that spun meshes also decreased over the course of the day (test for equality of proportions $\chi_{5}^{2}$ = 22.7, *p* < 0.001; chi-squared test for trend in proportions, $\chi_{1}^{2}$ = 16.6, *p* < 0.001, Figure 7).

## 4. Conclusions

The results obtained from the artificial burrow field trial imply that during the summer months, the absence of trapdoors has a marginal effect on the thermal environment at the bottom of “*L. hyraculus*” burrows and probably do not affect spider behavior. In addition, the hydric environment was unaffected, and we therefore conclude that under the weather conditions that this study was done, the conditions at the bottom of the burrows were essentially decoupled from the above-ground airflow. The fact that most *“L. hyraculus"* do not attempt to cover their burrow entrances immediately upon trapdoor removal at midday in the summer lends support to this conclusion. In this light, the trapdoor’s roles are more likely predator and parasite avoidance and prevention of flooding, and debris from falling in.

## Data Availability

All raw data is available upon request from berliner@bgu.ac.il.
